# The Role of EUS-BD in the Management of Malignant Biliary Obstruction: The Indonesian Perspective

**DOI:** 10.1155/2017/4856276

**Published:** 2017-10-18

**Authors:** Dadang Makmun, Achmad Fauzi, Murdani Abdullah, Ari Fahrial Syam

**Affiliations:** Division of Gastroenterology, Department of Internal Medicine, Faculty of Medicine, Universitas Indonesia, Cipto Mangunkusumo National General Hospital, Jakarta Pusat, Jakarta 10430, Indonesia

## Abstract

**Aims:**

To evaluate the success rate and related factors of endoscopic ultrasound guided-biliary drainage (EUS-BD).

**Material and Methods:**

We conducted a retrospective study among 24 patients with malignant biliary obstruction who underwent EUS-BD after failed ERCP from January 2015 to December 2016 in a tertiary health center. The bilirubin levels before and after the procedure were used to define the clinical success rate, while the stent deployment was used to define the technical success rate. We placed either transluminal biliary stents or transpapillary biliary stents.

**Results:**

Among 24 patients, choledochoduodenostomy technique was conducted in 23 patients (95.8%) and hepaticogastrostomy technique in 1 patient (4.2%). Transluminal stent placement was conducted in 23 patients, while transpapillary stent placement was conducted in 1 patient. The clinical success rate was 78.2% (18) in choledochoduodenostomy route and 100% (1) in hepaticogastrostomy route. EUS-BD was 2.37 times and 2.11 times more likely to be successful in reducing the bilirubin level in patients with tumor of the head of pancreas and periampullary tumor, respectively, but not in cholangiocarcinoma.

**Conclusions:**

EUS-BD is an effective and efficient procedure to achieve biliary drainage among patients with malignant biliary obstruction after ERCP failure.

## 1. Introduction

Obstructive jaundice occurs when there is an obstruction to the passage of bile from the liver to the duodenum. The cause of this condition varies from benign conditions to malignant conditions in the biliary system. Obstructive jaundice is not a definitive diagnosis; it requires additional examinations and diagnostic procedures to determine the primary disease. The mortality and morbidity of obstructive jaundice depend on the cause of the obstruction [1].

Currently, endoscopic retrograde cholangiopancreatography (ERCP) is the main therapeutic procedure used in the management of biliary obstruction. For many decades, percutaneous transhepatic biliary drainage (PTBD) and surgical interventions have been the alternative procedures when ERCP failed to achieve biliary drainage, but those methods have been related to high-risk complications and prolonged hospitalization [2, 3].

Endoscopic ultrasonography (EUS), which was developed in the early 1980s, has become a valuable imaging modality to visualize the gastrointestinal luminal wall and its surrounding structures, including the pancreatobiliary system. The large channel echo endoscopes of EUS have allowed for the therapeutic application of EUS by combining real-time ultrasound and fluoroscopy imaging of ERCP to carry out an EUS-biliary drainage (EUS-BD). EUS-BD can be used as a less invasive alternative procedure instead of PTBD and surgical intervention. The superiority of EUS-BD compared to ERCP and PTBD includes the possibility of accessing the bile duct system through many routes. Wiersema et al. were the first to report on EUS-guided diagnostic cholangiography in 1996, and in 2001 Giovannini et al. reported the first EUS-BD using transduodenal stenting to create a choledochoduodenal fistula with a needle knife. After this initial report, many studies reported modifications of the techniques and expanding indications for EUS-BD. EUS is relatively new in Indonesia and it was firstly introduced in our hospital, Cipto Mangunkusumo National General Hospital, a top national referral hospital, in 2013. In 2015 our experienced endoscopists started to develop EUS-BD after being trained from several endoscopy training centers in Asia such as India and Thailand. Until today, only seven among more than 2,000 hospitals in our country provide EUS facilities. For EUS-BD procedure, the most frequently used stent is plastic stent due to the price and its availability. Recently, EUS-BD is one of the treatments of choice to perform biliary drainage when ERCP has failed [2, 4–7].

EUS-BD techniques are divided into two categories, a rendezvous technique and a direct access technique. In the rendezvous technique for EUS-BD, a temporary fistula at the stomach or the bulb is created followed by placement of a guidewire through the biliary duct and the ampulla into the duodenum; then, ERCP is reperformed using the EUS-guided placed wire. The direct access technique includes EUS-guided choledochoduodenostomy (EUS-CDS), EUS-guided hepaticogastrostomy (EUS-HGS), and EUS-guided gallbladder drainage (EUS-GBD) [8, 9].

This study aimed to retrospectively evaluate the primary diseases, access route, and success rate of stent placement and related factors among patients with malignant biliary obstruction who underwent EUS-BD after failed ERCP at a top national referral hospital.

## 2. Materials and Methods

### 2.1. Patient Characteristics

This study was conducted among patients with malignant biliary obstruction who underwent EUS-BD after failed ERCP from January 2015 to December 2016 (*n* = 24) at a top national referral hospital. We collected data from 755 patients who underwent ERCP, and 241 among them were diagnosed with malignant biliary obstruction such as tumor of head of pancreas, periampullary tumor, cholangiocarcinoma, and Klatskin tumor. From the 241 patients, we excluded patients who had a successful ERCP procedure, resulting in 24 patients who required an alternative technique for biliary drainage due to failed ERCP.

All patients enrolled in this study were diagnosed as having inoperable malignant obstructive jaundice based on clinical symptoms (jaundice, dark-colored urine, and pale stool), laboratory examinations (elevated bilirubin levels, alkaline phosphatase levels, and gamma glutamyl transferase levels), and imaging examinations including transabdominal ultrasound, computed tomography (CT) scan of the abdomen, magnetic resonance cholangiopancreatography (MRCP), and EUS.

### 2.2. Access Routes and Drainage Route

The EUS-BD procedure was performed by experienced endoscopists who have performed over 100 EUS procedures. EUS-CDS was performed through the transluminal route from the duodenal bulb into the bile duct in 23 patients (transduodenal access), and EUS-HGS was performed through the transluminal route from the stomach into the bile duct in 1 patient (transhepatic access) where insertion of the scope into the duodenum failed.

**Figure 1:**
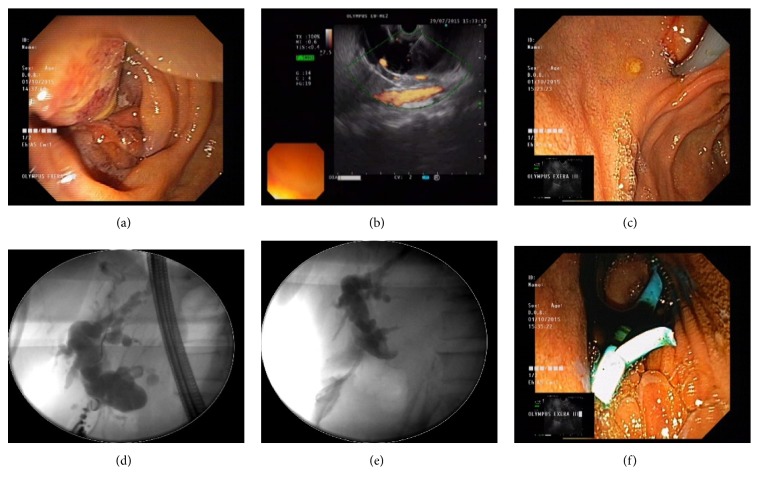
EUS-BD in a patient with a periampullary tumor with unidentifiable ampulla and failed ERCP: (a) endoscopy view of the periampullary tumor; (b) EUS imaging of the puncture into dilated CBD; (c) puncture point enlargement using Cystotome; (d) dilated intra- and extrahepatic CBD; (e) transduodenal plastic biliary stent placement; (f) bile drainage through the stent.

We used an Olympus GF type UCT 180 linear echoendoscope. The scope was introduced into the stomach or the duodenum. After visualization of the dilated bile duct, the scope was manipulated until an appropriate puncture route was identified. The stomach or the duodenal wall was then punctured with a 19-G needle (EchoTip Ultra; Cook Medical) into the bile duct. After that, a guidewire was inserted, and the fistula was dilated with an electrical needle knife (Zimmon papillotomy knife or Cystotome; Cook Medical) to facilitate stent placement. We placed a 10 Fr Cotton-Leung plastic stent in 21 patients and a fully covered self-expandable metallic stent (SEMS) with diameter of 10 mm in 3 patients. All stents were inserted in either an antegrade direction or retrograde direction (rendezvous technique). The insertion in the antegrade direction was performed after creating a tract from the stomach or duodenum into the bile duct, and the stent was placed following guidewire insertion. The insertion in retrograde direction was performed by inserting a guidewire into the bile duct and maneuvering it across the papilla into the duodenum. After that, the echoendoscope was withdrawn, leaving the guidewire. Then, the duodenoscope was inserted parallel to the guidewire until it was in front of the papilla. The guidewire was then caught with a snare through the working channel of the duodenoscope, and finally the stent was placed using a conventional ERCP procedure. The procedure of EUS-BD in a patient with a periampullary tumor is shown in Figure 1.

The results of the EUS-BD procedures were assessed using the technical success rate and clinical success rate. The technical success rate was defined as the success of deployment of the stent at the end of the procedure. The clinical success rate was defined as a 30% decrease in the bilirubin level one week after the procedure [10].

### 2.3. Data Collection

We retrospectively collected the data of the enrolled patients by reviewing the medical records of our hospital, including demographic data (age, gender, and body mass index (BMI)), EUS-BD technique (hepaticogastrostomy or choledochoduodenostomy), laboratory data (bilirubin levels, alkaline phosphatase levels, and gamma glutamyl transferase levels), primary disease (tumor of head of pancreas, periampullary tumor, or cholangiocarcinoma), EUS-BD indication, and complications. The collected data were then classified based on published literature to make them easier to analyze.

The patients were divided into groups based on demographic data such as age and BMI. The patients were divided by age in the group < 60 years and the group ≥ 60 years, while for the BMI data we used the World Health Organization (WHO) recommendation for Asian criteria of BMI classification to interpret the results. The patients were divided into those with difficult cannulation and those with unidentifiable ampulla based on the EUS-BD indication data. The laboratory data included the bilirubin level before the procedure and one week after the procedure. Complications that were observed included all complications that occurred during or after the EUS-BD procedure, such as bleeding, perforation, pneumoperitoneum, cholangitis, bile leakage, and death.

### 2.4. Statistical Analysis

All collected data were analyzed using univariate analysis to evaluate the correlation between the independent variables and dependent variables. The independent variables included age, gender, BMI, primary disease, EUS-BD indication, and EUS-BD complications. The dependent variable was the clinical success rate of all enrolled patients.

The Chi-square test method was used to examine the statistical significance of the correlation between dependent and independent variables. The required conditions for the Chi-square tests were that the value of the expected count inside the 2 × 2 table should be more than 5, and if the value was less than 5, we used the Fisher's exact test. The result was considered significant when *p* value < 0.05, and if the *p* value > 0.05, it was considered to be insignificant. Data analysis was done using SPSS computer software version 23.0 (version 23.0; IBM SPSS, IBM Corp., Armonk, NY, USA).

## 3. Results

Among the 24 patients who underwent EUS-BD, 54.2% were male, and 45.8% were female. The age of the patients ranged from 37 to 80 years with 54.2% aged < 60 years and 45.8% aged ≥ 60 years. According to BMI criteria, 29.2% were underweight, 45.8% were normoweight, and 25% were preobese. Primary diseases affecting the patients included tumor of the head of pancreas (54.2%), periampullary tumor (41.6%), and cholangiocarcinoma (4.2%). We also found that the most widely used EUS-BD technique was choledochoduodenostomy (95.8%). The indication of EUS-BD among all patients included difficult cannulation (66.7%) and unidentifiable ampulla (33.3%). Among 23 patients who underwent EUS-CDS, 20 received plastic stent and 3 received fully covered SEMS. One patient who underwent EUS-HGS received plastic stent. Stent placement in all patients was facilitated with electrical needle knife (Cystotome). Complications were developed in 4 patients (16.7%). Pneumoperitoneum caused by perforation during the procedure was found in 1 patient, and cholangitis was found in 3 patients. As a result, all 4 patients died within one week after the procedure. The causes of death included general peritonitis which occurred shortly after perforation and septic shock due to cholangitis. The characteristic of all subjects is summarized in Table 1.

In this study, stent placement could be achieved among patients who underwent EUS-BD procedure. The analysis showed that the technical success rate for both techniques of EUS-CDS and EUS-HGS was 100%. Based on the 30% bilirubin level reduction one week after the procedure, the clinical success rate for both techniques was 79.1%. The clinical success rates for the choledochoduodenostomy approach and the hepaticogastrostomy approach were 78.2% and 100%, respectively. One week after the procedure, 19 patients achieved successful biliary drainage, and the remaining 5 patients were unable to achieve biliary drainage. The outcome of EUS-BD procedures is described in Table 2. Among the 4 patients who died, 2 showed a decrease in the bilirubin level of more than 30% within one week after the procedure, indicating successful biliary drainage. The bilirubin level of all subjects before and after the procedure is shown in Table 3.

Statistical analysis showed that the demographic data of the patients including gender, age, and BMI had no significant correlation with the clinical success rate of EUS-BD, *p* value > 0.05 (Table 4).

We found that EUS-BD was 2.37 times and 2.11 times more likely to be successful in reducing the bilirubin level in patients with tumor of the head of the pancreas and periampullary tumor, respectively. There was no association between clinical success rate of EUS-BD and cholangiocarcinoma (RR = 1, CI 0.949–1.174). In this study, the clinical success rate of EUS-BD did not reach statistical significance due to a small sample size. Only tumor of the head of pancreas and periampullary tumor had *p* value < 0.05. Factors related to the clinical success rate of EUS-BD are described in Table 5.

## 4. Discussion

This study found that patients with malignant biliary obstruction who required the EUS-BD procedure after failed ERCP were more often male than female, with the proportions being 54.2% and 45.8%, respectively. Similar results were reported in the study by Park et al. and Khashab et al. Park et al. reported that, among the 57 patients enrolled in their study, 35 patients (60.3%) were male [11]. Another multicenter prospective study involving 12 tertiary centers by Khashab et al. showed that, among the 96 patients enrolled in the study, 53 patients were male (55%) [12]. As of yet, there is no explanation regarding the tendency of malignant biliary obstructions that require a EUS-BD procedure after failed ERCP to affect males more than females. This topic requires more specific research with a larger sample population to evaluate the correlation between gender and the incidence of malignant biliary obstructions that require alternative biliary drainage after failed ERCP.

When examining age, our study found that the incidence of malignant biliary obstructions that required a EUS-BD procedure was more common in patients < 60 years (54.2%) than in patients ≥ 60 years (45.8%), with a mean age of 59 years. Another study by Khashab et al. reported that the incidence of malignant biliary obstructions that required EUS-BD occurred at a mean age of 66 years, while Kawakubo et al. found that the incidence of this circumstance occurred at a mean age of 72 years. Both of these studies revealed that the older age group is more commonly affected by malignant biliary obstructions that ultimately require EUS-BD for bile drainage after ERCP failure [10, 11]. Compared to the other studies by Khashab et al. and Kawakubo et al., the present study revealed a slight tendency of malignant biliary obstructions requiring EUS-BD to affect younger patients, with a mean age of 59 years. This discrepancy might be caused by the sample size, which in our study was smaller than that in the other studies. The present study was conducted in a tertiary center with 24 patients. In contrast, Khashab et al. conducted their study in 12 tertiary centers with 96 enrolled patients, and Kawakubo et al. also conducted their study in multiple centers with 64 enrolled patients [12, 13]. Another reason for this discrepancy is the different age range of patients in each study. The study by Kawakubo et al. included patients in the age range of 66–79 years, while our study included patients in the age range of 37–80 years, therefore resulting in a different mean age. Similar to this situation, a study conducted by Makmun et al. in Indonesia showed that there is a changing trend of gastrointestinal malignancy affecting people at a younger age, especially in the age group of 40–60 years [14].

The statistical analysis showed that there was no correlation between demographic characteristics and the clinical success rate of EUS-BD. Gender, as one of the analyzed variables, showed no significant correlation with the clinical success rate of EUS-BD (*p* value = 0.590). We also found no significant correlation with age or BMI (*p* value = 0.590, *p* value = 0.586, resp.). Hence, from the statistical analysis, there was no correlation between the clinical success rate of EUS-BD and gender, age, or BMI.

In a case report from Obana and Yamasaki of an obese patient (BMI: 35.1 kg/m^2^) who underwent EUS-BD with the indication of difficult cannulation during ERCP, the bilirubin level clearly decreased from 14.1 mg/dL (before the procedure) to 2.3 mg/dL (after the procedure). The authors reported that EUS-BD could be performed successfully in this obese patient to achieve biliary drainage [15]. There have been no reports regarding the correlation of age or gender with the clinical success rate of the EUS-BD procedure.

The most common primary disease in our study was tumor of the head of pancreas (54.2%), and the remaining primary diseases were periampullary tumor (41.6%) and cholangiocarcinoma (4.2%). Other studies reporting similar findings include those conducted by Dhir et al., Khashab et al., and Kawakubo et al. These studies also found that tumor of the head of pancreas was the most common diagnosis in malignant biliary obstructions that require the EUS-BD procedure [7, 12, 13]. On the other hand, a study conducted by Panpimanmas and Ratanachu-Ek in Thailand found that the most common diagnosis in malignant biliary obstructions that required EUS-BD was cholangiocarcinoma [16]. Another report by Kuberan et al. found that periampullary carcinoma was more common than carcinoma of the head of pancreas as a cause of malignant biliary obstructions that required EUS-BD after failed ERCP [17]. The various results of the abovementioned studies need to be investigated thoroughly. The differences in study methods and sample size might have contributed to the discordant results.

In this study, tumor of the head of pancreas and periampullary tumor are 2.37 times and 2.11 times more likely to be successful in reducing bilirubin level after EUS-BD. In patient with cholangiocarcinoma, the likelihood of EUS-BD clinical success rate was 1.05. These results might be due to the involvement of the proximal biliary tract among patients with cholangiocarcinoma, which causes difficulty in achieving biliary drainage compared to patients with tumor of the head of pancreas and periampullary tumor. Currently, no other published studies have evaluated the correlation between the primary disease and the clinical success rate among patients with malignant biliary obstruction who required EUS-BD as an alternative for bile drainage after failed ERCP.

Another analyzed related factor in this study was the EUS-BD technique used. We used two EUS-BD techniques in this study (EUS-CDS and EUS-HGS), resulting in a 79.1% (19/24) clinical success rate of both techniques. The EUS-CDS technique was carried out in 23 patients, and this technique had technical and clinical success rates of 100% (23/23) and 78.2% (18/23), respectively.

This result is inconsistent with the study conducted by Ikeuchi and Itoi, which reported that the average technical success rate of EUS-CDS was 91.8% (312/348) and the clinical success rate was 94.5% (223/236) [18]. Another review article by Artifon reported that the success rate of EUS-BD among patients with malignant biliary obstruction was above 70% [19]. A study conducted by Ogura and Higuchi involving more than 200 patients who underwent EUS-HGS reported that the technical and clinical success rates ranged from 65% to 100% and 87% to 100%, respectively [20]. On the other hand, Ikeuchi et al. reported that the average technical success rate of EUS-HGS was 95.4% (146/153), and the clinical success rate was 90.9% (100/110) [18, 19]. There have been no recent guidelines for the selection of EUS-BD techniques. Many endoscopists use EUS-CDS as their first choice of procedure due to the lower rate of complications compared to EUS-HGS [18, 21]. Therefore, the selection of the EUS-BD procedure is based on the patient's condition (patients with gastric obstruction, the site of the biliary obstruction, and patients with Roux-en-Y anastomosis), availability of equipment, and the decision of the endoscopist [21, 22]. Several studies have reported that there is no significant difference in the success rate, clinical success rate, or complications between EUS-CDS and EUS-HGS [21, 23, 24]. However, a retrospective multicenter study conducted by Dhir et al. found that complications from EUS-BD were more common in EUS-HGS than EUS-CDS [7].

Electrical needle knife (Cystotome), catheter dilator, and balloon dilator could be used to facilitate stent placement during EUS-BD procedures. In our study the stent placement could be facilitated with Cystotome only. Among 23 patients who underwent EUS-CDS, plastic stents with the diameter of 10 Fr were placed in 20 patients, and in the remaining 3 patients we used fully covered SEMS with diameter of 10 mm. Our study used more plastic stents than fully covered SEMS due to the price of the stent and the availability at the time the procedure was performed. In 5 patients, stent placement could not decrease the bilirubin level by 30% within a week after the procedure. It might be due to clogging of the stent or tumor infiltration. In our study, EUS-CDS could not be performed in 1 patient due to tumor ingrowth into the duodenal bulb which caused the failure of the scope insertion into the duodenum. Hence, the endoscopists used EUS-HGS as an alternative procedure. Based on the experience in our study, EUS-CDS could be used as the first method of choice to be performed among patients who required EUS-BD after failed ERCP.

In terms of EUS-BD indication, our study showed two indications, including difficult cannulation (66.7%) and unidentifiable ampulla (33.3%). The EUS-BD indication resulting from this study was different than those from another study conducted by Kim et al., which showed EUS-BD indications including periampullary tumor obstruction as the most common EUS-BD indication, followed by difficult cannulation, high-grade stricture, high-grade left-sided hilar stricture, duodenal stenosis due to a previous duodenal ulcer, and tumor obstruction of the duodenum [25].

Our study also reported complications that occurred after the procedure, including pneumoperitoneum (1 patient) and cholangitis (3 patients). A review article by Ikeuchi and Itoi involving 38 studies reported that the most common complication of EUS-CDS procedures was peritonitis, and other complications included pneumoperitonitis, bleeding, bile leakage, perforation, abdominal pain, biloma, cholangitis, pancreatitis, hemobilia, and stent misplacement. The other 21 studies reported that the complications associated with EUS-HGS included cholangitis, bleeding, stent migration, biloma, bile leakage, pneumoperitonitis, peritonitis, stent misplacement, abdominal pain, metal stent shrinkage, and ileus [18]. In our study, among all patients who underwent EUS-BD there was no case of biliary leak. We cannot evaluate the correlation between the techniques used and the complications occurred during or after the procedure, because almost all enrolled patients underwent EUS-CDS and only 1 patient underwent EUS-HGS.

## 5. Conclusions

In conclusion, patients with tumor of the head of pancreas and periampullary tumor had higher clinical success rates for EUS-BD than patients with cholangiocarcinoma among those with malignant biliary obstructions that required EUS-BD after failed ERCP. There was no correlation between the clinical success rate of EUS-BD and the patients' demographic characteristics, EUS-BD technique, or EUS-BD indication. When the ERCP procedures fail, EUS-BD is an effective and efficient procedure to achieve biliary drainage, and EUS-CDS could be used as the first method of choice.

## Figures and Tables

**Table 1:** Patient characteristics.

Characteristics	Frequency (*n*)	Percentage (%)
*Gender*		
Male	13	54.2
Female	11	45.8
*Age (mean ± 59)*		
<60 years	13	54.2
≥60 years	11	45.8
*BMI (mean ± 20.36, median ± 19.25)*		
Underweight	7	29.2
Normal	11	45.8
Preobese	6	25
*Primary disease*		
Tumor of the head of pancreas	13	54.2
Periampullary tumor	10	41.6
Cholangiocarcinoma	1	4.2
*Technique*		
Choledochoduodenostomy (CDS)	23	95.8
Hepaticogastrostomy (HGS)	1	4.2
*EUS-BD indication*		
Difficult cannulation	16	66.7
Unidentifiable ampulla	8	33.3
*Stents*		
Plastic stent	21	87.5
SEMS	3	12.5
*Complications*		
Cholangitis	3	12.5
Pneumoperitoneum	1	4.1

**Table 2:** Outcome of EUS-BD procedures.

Outcome	Frequency (*n*)	Percentage (%)
*Technical success*		
Choledochoduodenostomy	23/23	100
Hepaticogastrostomy	1/1	100
*Clinical success*		
Choledochoduodenostomy	18/23	78.2
Hepaticogastrostomy	1/1	100
*Overall clinical outcome*		
Successful	19/24	79.1
Unsuccessful	5/24	20.9

**Table 3:** Patient characteristics and bilirubin levels before and after the procedure.

Patient	Gender	Age (years)	BMI	Primary disease	Total/direct bilirubin (TB/DB) before procedure (mg/dL)	Total/direct bilirubin (TB/DB) one week after the procedure (mg/dL)
(1)	Male	73	27.3	Periampullary tumor	25.75/22.4	8.71/7
(2)	Male	43	17.3	Tumor of head of pancreas	24.46/20.27	18.72/15.53
(3)	Female	58	21.4	Tumor of head of pancreas	37.85/29.85	13.22/11.71
(4)	Female	66	10.5	Periampullary tumor	16.02/13.99	10.86/9.42
(5)	Male	75	28.51	Tumor of head of pancreas	30.02/24.45	14.75/12.87
(6)	Male	77	25.71	Tumor of head of pancreas	18.25/15.65	14.41/10.89
(7)	Male	54	18.37	Tumor of head of pancreas	3.89/2.8	1.41/1.09
(8)	Female	63	15.62	Periampullary tumor	23.87/20.23	15.83/14,46
(9)	Female	63	18.73	Tumor of head of pancreas	9.9/9.3	13.9/12.79
(10)	Male	41	19.5	Tumor of head of pancreas	20.6/18.8	15.06/13.52
(11)	Female	76	17.5	Tumor of head of pancreas	15.28/14.53	13.75/12.33
(12)	Female	43	18.7	Periampullary tumor	13.88/12.90	4.45/3.98
(13)	Female	59	19	Periampullary tumor	14.21/12.48	10.11/8.35
(14)	Female	78	21.22	Periampullary tumor	21/18	4.67/3.23
(15)	Female	64	25.7	Periampullary tumor	9.15/8.06	6.4/5.3
(16)	Male	54	19.92	Tumor of head of pancreas	18.4/17.06	9.11/5.8
(17)	Male	37	17.3	Periampullary tumor	8.7/6.6	6.03/4.79
(18)	Male	58	19.53	Periampullary tumor	29.4/21.75	21.53/19.08
(19)	Male	63	21.30	Periampullary tumor	22.06/19.91	3.6/3.5
(20)	Male	53	25.34	Tumor of head of pancreas	25.64/23.08	20.22/17.42
(21)	Male	46	17.93	Tumor of head of pancreas	29.32/22.75	6.63/5.58
(22)	Female	80	18.73	Cholangiocarcinoma	29.13/25.83	16.74/15.34
(23)	Female	45	26.03	Tumor of head of pancreas	28.26/25.4	14.08/12.1
(24)	Male	52	17.5	Tumor of head of pancreas	13.69/11.02	11.36/9.52

**Table 4:** Correlation between demographic data and the clinical success rate of EUS-BD.

Variables	Clinical success rate				
	*N*	%	RR	95% CI	*p* value
*Gender*					
Male	13	54.2	1.140	0.496–2.623	0.585
Female	11	45.8	0.844	0.261–2.730	1.0
*Age*					
<60 years	13	54.2	0.091	0.221–2.160	0.415
≥60 years	11	45.8	1.425	0.586–3.466	0.630
*BMI*					
Underweight	7	29.2	1.520	0.410–5.639	0.462
Normoweight	11	45.8	0.380	0.063–2.309	0.215
Preobese	6	25	1.900	0.477–7.569	0.366

**Table 5:** Factors related to the clinical success rate of EUS-BD.

Variables	Clinical success rate				
	*N*	%	RR	95% CI	*p* value
*Primary disease*					
Tumor of the head of pancreas	13	54.2	2.375	1.402–4.025	0.030
Periampullary tumor	10	41.6	2.111	1.314–3.391	0.047
Cholangiocarcinoma	1	4.2	1.056	0.949–1.174	0.792
*Technique*					
Choledochoduodenostomy	23	95.8	0.739	0.580–0.942	0.750
Hepaticogastrostomy	1	4.2	1.353	1.061–1.725	1.0
*EUS-BD indication*					
Difficult cannulation	16	66.7	1.0	0.613–1.632	0.698
Unidentifiable ampulla	8	33.3	1.0	0.230–4.349	1.0
